# Supplementary material for: Modeling glioblastoma heterogeneity as a dynamic network of cell states
Source: Mol Syst Biol. 2021 Sep 16;17(9):e10105. doi: 10.15252/msb.202010105 (PMC8444284; doi:10.15252/msb.202010105)
Supplement: Supplementary file 5 — Source Data for Figure 3 [file MSB-17-e10105-s001.zip › Figure3A_sourcedata/GSEA_3065/nosigdb_state6.GseaPreranked.1622718443993/RICHARDS_NEFTEL_CELL_2019_NPC2.html]

Details for gene set RICHARDS\_NEFTEL\_CELL\_2019\_NPC2[GSEA]

|  || Dataset | state6 |
| Phenotype | NoPhenotypeAvailable |
| Upregulated in class | na\_pos |
| GeneSet | RICHARDS\_NEFTEL\_CELL\_2019\_NPC2 |
| Enrichment Score (ES) | 0.5294416 |
| Normalized Enrichment Score (NES) | 1.7720653 |
| Nominal p-value | 0.0 |
| FDR q-value | 0.006725296 |
| FWER p-Value | 0.116 |
Table: GSEA Results Summary

  

Fig 1: Enrichment plot: RICHARDS\_NEFTEL\_CELL\_2019\_NPC2      
 Profile of the Running ES Score & Positions of GeneSet Members on the Rank Ordered List

  

| PROBE | GENE SYMBOL | GENE\_TITLE | RANK IN GENE LIST | RANK METRIC SCORE | RUNNING ES | CORE ENRICHMENT || 1 | SOX4 |  |  | 3 | 0.375 | 0.1516 | Yes |
| 2 | SOX11 |  |  | 70 | 0.230 | 0.2373 | Yes |
| 3 | KIF5C |  |  | 415 | 0.134 | 0.2522 | Yes |
| 4 | CEP170 |  |  | 452 | 0.128 | 0.2997 | Yes |
| 5 | DPYSL3 |  |  | 464 | 0.127 | 0.3498 | Yes |
| 6 | NDRG4 |  |  | 601 | 0.114 | 0.3804 | Yes |
| 7 | CD24 |  |  | 674 | 0.108 | 0.4159 | Yes |
| 8 | LBH |  |  | 695 | 0.106 | 0.4567 | Yes |
| 9 | PAK3 |  |  | 845 | 0.095 | 0.4782 | Yes |
| 10 | PKIA |  |  | 1063 | 0.082 | 0.4867 | Yes |
| 11 | RND3 |  |  | 1213 | 0.075 | 0.5000 | Yes |
| 12 | TCF4 |  |  | 1234 | 0.074 | 0.5278 | Yes |
| 13 | DPYSL5 |  |  | 1555 | 0.062 | 0.5164 | Yes |
| 14 | STMN1 |  |  | 1649 | 0.058 | 0.5294 | Yes |
| 15 | NFIX |  |  | 2055 | 0.046 | 0.5019 | No |
| 16 | MLLT11 |  |  | 2335 | 0.038 | 0.4853 | No |
| 17 | SEPT3 |  |  | 2710 | 0.029 | 0.4542 | No |
| 18 | NFIB |  |  | 2762 | 0.028 | 0.4596 | No |
| 19 | MAP1B |  |  | 2893 | 0.024 | 0.4545 | No |
| 20 | DDAH2 |  |  | 3061 | 0.021 | 0.4438 | No |
| 21 | NREP |  |  | 3707 | 0.007 | 0.3729 | No |
| 22 | BLCAP |  |  | 3962 | 0.001 | 0.3445 | No |
| 23 | CD200 |  |  | 4124 | -0.002 | 0.3268 | No |
| 24 | MIAT |  |  | 5393 | -0.026 | 0.1928 | No |
| 25 | TAGLN3 |  |  | 5582 | -0.030 | 0.1835 | No |
| 26 | DBN1 |  |  | 5587 | -0.030 | 0.1953 | No |
| 27 | ENO2 |  |  | 5889 | -0.036 | 0.1754 | No |
| 28 | RBFOX2 |  |  | 5896 | -0.036 | 0.1893 | No |
| 29 | TUBB2A |  |  | 7078 | -0.067 | 0.0817 | No |
| 30 | FNBP1L |  |  | 7173 | -0.070 | 0.0994 | No |
| 31 | UCHL1 |  |  | 7848 | -0.098 | 0.0624 | No |
| 32 | ATP1B1 |  |  | 8091 | -0.113 | 0.0804 | No |
Table: GSEA details [plain text format]

  

Fig 2: RICHARDS\_NEFTEL\_CELL\_2019\_NPC2: Random ES distribution      
 Gene set null distribution of ES for **RICHARDS\_NEFTEL\_CELL\_2019\_NPC2**
